# Functional QTL mapping and genomic prediction of canopy height in wheat measured using a robotic field phenotyping platform

**DOI:** 10.1093/jxb/erz545

**Published:** 2020-02-25

**Authors:** Danilo H Lyra, Nicolas Virlet, Pouria Sadeghi-Tehran, Kirsty L Hassall, Luzie U Wingen, Simon Orford, Simon Griffiths, Malcolm J Hawkesford, Gancho T Slavov

**Affiliations:** 1 Department of Computational & Analytical Sciences, Rothamsted Research, Harpenden, UK; 2 Department of Plant Sciences, Rothamsted Research, Harpenden, UK; 3 John Innes Centre, Norwich Research Park, Colney Lane, Norwich, UK; 4 Scion, Rotorua, New Zealand; 5 CSIRO Agriculture and Food, Australia

**Keywords:** Data smoothing, dimensionality reduction, dynamic QTLs, factor-analytic model, function-valued traits, genomic selection, phenomics

## Abstract

Genetic studies increasingly rely on high-throughput phenotyping, but the resulting longitudinal data pose analytical challenges. We used canopy height data from an automated field phenotyping platform to compare several approaches to scanning for quantitative trait loci (QTLs) and performing genomic prediction in a wheat recombinant inbred line mapping population based on up to 26 sampled time points (TPs). We detected four persistent QTLs (i.e. expressed for most of the growing season), with both empirical and simulation analyses demonstrating superior statistical power of detecting such QTLs through functional mapping approaches compared with conventional individual TP analyses. In contrast, even very simple individual TP approaches (e.g. interval mapping) had superior detection power for transient QTLs (i.e. expressed during very short periods). Using spline-smoothed phenotypic data resulted in improved genomic predictive abilities (5–8% higher than individual TP prediction), while the effect of including significant QTLs in prediction models was relatively minor (<1–4% improvement). Finally, although QTL detection power and predictive ability generally increased with the number of TPs analysed, gains beyond five or 10 TPs chosen based on phenological information had little practical significance. These results will inform the development of an integrated, semi-automated analytical pipeline, which will be more broadly applicable to similar data sets in wheat and other crops.

## Teaser

Functional analysis of longitudinal phenotypic data for five and 10 time points saturates QTL detection power and genomic predictive ability for canopy height in wheat.

## Introduction

In recent years, field-based high-throughput plant phenotyping (HTPP) tools have been used extensively in crop trials, aiming to reduce or eliminate manual measurements, increase the amount and quality of data for temporally dynamic phenotypes, and, ultimately, translate ‘big data’ collected using various sensors into knowledge (Rebetzke *et al.*, 2016; Sadeghi-Tehran *et al.*, 2017; Virlet *et al.*, 2017; Araus *et al.*, 2018). However, even with rapidly increasing computational power and efficiency, image post-processing (i.e. alignment, calibration, and segmentation) is demanding, and the collection of data throughout the crop growth cycle of large trials remains onerous (Araus and Cairns, 2014; Haghighattalab *et al.*, 2016; Pauli *et al.*, 2016). Thus, collecting measurements at excessively frequent intervals may not be cost-efficient (Araus and Kefauver, 2018; Reynolds *et al.*, 2019) and can even result in poorer overall quality of the data set, as certain phenological stages (e.g. reproductive) are particularly prone to data collection errors (Dreccer *et al.*, 2019; Rebetzke *et al.*, 2019). Because of these limitations and the critical importance of manual or automated data cleaning (Tardieu *et al.*, 2017), the number of time points (TPs) for which HTPP data are to be extracted and analysed is an important consideration, as is the distribution of these data TPs throughout the growing period of the crop (Rutkoski *et al.*, 2016; Dreccer *et al.*, 2019).

The opportunities created by the availability of HTPP data also pose significant analytical challenges in genetic studies aimed at identifying quantitative trait loci (QTLs) in mapping families (Li and Sillanpaa, 2015; Sun and Wu, 2015) and genomic prediction of complex phenotypic traits in breeding populations (Cabrera-Bosquet *et al.*, 2012; van Eeuwijk *et al.*, 2019). Functional mapping based on maximum likelihood (Ma *et al.*, 2002) was originally proposed to enhance conventional QTL mapping approaches by capturing the additional dynamic information in longitudinal phenotypic data using mathematical functions [i.e. treating time series as ‘function-valued traits’ (Stinchcombe *et al.*, 2012)]. Over the last two decades, this methodology has been applied across a range of experimental systems (Wu and Lin, 2006; Li *et al.*, 2010; Jiang *et al.*, 2015; Xu *et al.*, 2016). However, when implemented in its original version, functional mapping is computationally inefficient, particularly when large numbers of TPs and/or markers are analysed (Wang *et al.*, 2017). In an attempt to overcome these limitations, while retaining or further increasing statistical power, Moore *et al.* (2013) and Kwak *et al.* (2014) proposed more computationally efficient regression-based methods. These approaches were refined further by Kwak *et al.* (2016), who replaced the original observed trait data with a smoothed approximation (i.e. reducing phenotype noise) and then applied a dimensionality reduction technique to reduce the number of tests.

Previous studies of wheat plant height, an important agronomic trait, have identified several dynamic QTLs (Wang *et al.*, 2010; Wu *et al.*, 2010; Zhang *et al.*, 2017). In addition, functional mapping approaches have generated empirical evidence that QTLs can be expressed (i) during very short periods (hereafter referred to as ‘transient’ QTLs); (ii) during specific phenological stages; or (iii) throughout the growing season (hereafter referred to as ‘persistent’ QTLs) (Bac-Molenaar *et al.*, 2015; Al-Tamimi *et al.*, 2016; Campbell *et al.*, 2017; Feldman *et al.*, 2017; Muraya *et al.*, 2017; Ward *et al.*, 2019). Recently, Camargo *et al.* (2018) used longitudinal data from a glasshouse HTPP facility to characterize several physiological and morphometric wheat traits in much greater detail. However, similar mapping studies of wheat grown under field conditions are currently lacking.

Several decades of linkage mapping studies have resulted in the identification of thousands of QTLs across a wide variety of traits and crops, but the rate of translation of these QTLs into marker-assisted selection in breeding programmes has generally been very poor (Bernardo, 2016; Wallace *et al.*, 2018). With the gradual realization that genomic selection is the only viable option of using marker–trait associations in breeding programmes (Crossa *et al.*, 2017), the interest of geneticists working with HTPP function-valued data is increasingly shifting towards genomic prediction (Rutkoski *et al.*, 2016; Aguate *et al.*, 2017; Campbell *et al.*, 2017, 2018; Montesinos-Lopez *et al.*, 2017a, b, 2018; Sun *et al.*, 2017, 2019; Watanabe *et al.*, 2017; Crain *et al.*, 2018; Juliana *et al.*, 2019; Krause *et al.*, 2019; van Eeuwijk *et al.*, 2019). However, despite several methodological developments in this field, many questions remain. For example, it is not currently clear if increasing the number of TPs consistently helps to increase the predictive ability for temporally dynamic traits, or how to most efficiently extract simple, genetically meaningful parameters from highly dimensional data (van Eeuwijk *et al.*, 2019). Most studies so far have focused on multitrait and random regression models (Sun *et al.*, 2017; Campbell *et al.*, 2018; Crain *et al.*, 2018), but a number of potentially more effective approaches (e.g. functional regression analysis) have not yet been fully explored (Montesinos-Lopez *et al.*, 2017a, 2018).

In this study, we used a high-throughput Field Scanalyzer (Virlet *et al.*, 2017) to collect a dense time series of height measurements in a wheat mapping population. Our goals were to (i) combine individual TP and functional mapping approaches for the detection of transient, stage-specific, and persistent QTLs; (ii) explore various approaches for using longitudinal data for genomic prediction; and (iii) compare results between different sampling intensities (i.e. number of TPs) and sampling strategies (i.e. systematically interspersed versus informed by phenology).

## Materials and methods

### Mapping population and field experiment

A recombinant inbred line (RIL) population (F_7_ generation) comprising 197 lines derived from crossing the Chinese Spring (CS) and Paragon (PAR) genotypes was used in this study (Allen *et al.*, 2017; Wingen *et al.*, 2017). The field trials were conducted on the Field Scanalyzer phenotyping platform (LemnaTec GmbH) (Virlet *et al.*, 2017) during the 2015–2016 (hereafter referred to as the 2016 data set) and 2016–2017 (hereafter referred to as the 2017 data set) growing seasons (from November to June) at Rothamsted Research, Harpenden, UK (51°48'34.56''N, 0°21'22.68''W). The experimental scheme was laid out in augmented blocks consisting of RILs, as well as one (2016; Crusoe) and five (2017; CS, PAR, Crusoe, and two RILs) check genotypes. Plots of 1.0×1.0 m were used in 2016 and 0.8×1.0 m in 2017. They were spaced 0.2 m apart, and the within-plot inter-row distance was 0.15 m. In 2016, RILs were grown in individual rows (six different RILs per plot) and, in 2017, RILs were grown in three consecutive rows (two RILs per plot). Both experiments were grown under conventional fertilization, weed, and pest control.

### High-throughput data collection and image processing

A fully automated, high-throughput, and fixed-site phenotyping platform was used to acquire all data (Virlet *et al.*, 2017). The twin lasers within the camera bay were used to collect data on multiple days throughout the growing season, from tillering through flowering (growth stages 20–69) (Zadoks, 1974; Tottman, 1987), and canopy height was extracted from the point clouds. Briefly, the main steps of the method consisted of merging the two point clouds, cropping the RIL of interest, and then splitting it into sub-regions (Supplementary Fig. S1 at *JXB* online, step 2 to 3). Subsequently, an average canopy height was estimated for each sub-region of the RIL and then averaged together to obtain the plot canopy height (Supplementary Fig. S1, step 4 and 5). A detailed description of the steps is given in the text preceding Supplementary Fig. S1. In total, data for 22 TPs [between 90 and 251 days after sowing (DAS)] for 2016 and 26 TPs (between 23 and 228 DAS) for 2017 were automatically extracted for each plot. In addition, canopy height of each RIL was measured manually on five representative plants at four TPs in 2016 and 2017 (Supplementary Fig. S2). For the latter data set, the coefficient of determination (*R*^2^) between manual and automatic measurements was 0.59, 0.78, 0.85, and 0.81 for 70, 155, 208, and 233 DAS, respectively (Supplementary Fig. S2B). However, we observed reduced data quality in the 2016 data set after 196 DAS due to a strong overlap between RILs as each row was planted with a different RIL (i.e. *R*^2^ of 0.66, 0.66, 0.35, and 0.55 for 115, 196, 216, and 239 DAS, respectively) (Supplementary Fig. S2C). Thus, we used the 2016 data set only for validation of the 2017 QTL mapping analyses.

### Phenotypic analysis

#### Factor-analytic (time series) analysis

We used the ASReml package (Butler *et al.*, 2009) within R (R Core Team, 2018) to obtain the best linear unbiased predictions (BLUPs) for the RIL genotypes across all TPs by fitting the following mixed linear model:

$$\begin{array}{l} y=X\beta \;+Cw+Zg+\varepsilon \; \end{array}$$ (1)

where ***y*** was a matrix of phenotypic values of genotypes across TPs; **β** was a vector of the fixed effects of TPs, ***w*** was a vector of the fixed effects of check genotypes, ***g*** was a vector of the random-effect genotypic values of individuals across TPs, and **ε** was a vector of random residuals. The incidence matrices for **β**, ***w***, and ***g*** were ***X***, ***C***, and ***Z***, respectively. A factor-analytic (FA, order 1) variance–covariance structure (VCOV) was used for the genotype effects (Meyer, 2009). Thus, we assumed ***g***~MVN(0,σ ^2^_g_⊗***I***), with covariance matrix σ ^2^_g_=Var(**g**)Σ⊗A, where Σ=ΛΛ ^T^ + Ψ (Λ was a *t* TP × *k* loadings matrix, and Ψ was a *t*×*t* diagonal matrix with a specific variance for each TP), A was the numerator relationship matrix (we used the ***I*** identity matrix), ⊗was the Kronecker product, and MVN was the multivariate normal distribution. For the residual term, we assumed heterogeneous variances between TPs (block-diagonal structure) jointly with a two-dimensional separable autoregressive (AR1) matrix to fit the row and column effects (i.e. spatial correction). Thus, the vector of errors was partitioned into **ε**=ξ+**η**, where ξ and **η** refer to the spatially correlated and independent errors, respectively. The variance of residuals was assumed to be $Var\left(\;\varepsilon \;\right)=var\left(\xi +\eta \;\right)=R=\Sigma_{\xi }^{2}\left[\sum_{c}\left(\Phi_{c}\right)⊗\sum_{r}\left(\Phi_{r}\right)\right]+\left(I⊗D_{\eta \;}\right)$, where σ ^2^_ξ_ was the variance due to local tendency and ***D***_**η**_ was a *t*×*t* diagonal VCOV matrix, with each TP having a specific spatially independent variance component. Matrices $\sum_{c}\left(\Phi_{c}\right)$ and $\sum_{r}\left(\Phi_{r}\right)$ formed the AR1 matrix with auto-correlation parameters Φ _c_ and Φ _r_ and order given by the number of columns (c) and rows (r), respectively (Gilmour *et al.*, 1997). The significance of random and fixed effects was estimated for each TP using likelihood ratio and Wald tests at α=0.01 using the reml.lrt.asreml and wald functions from the asremlPlus R (Brien, 2019) and ASReml-R packages, respectively (Supplementary Table S1).

In order to visualize genetic parameters, we generated a surface quadratic plot of covariance matrix of TPs as follows:

$\text{Var}\left(\text{g}\right)=\Sigma ⊗\text{A}=\left[\begin{array}{ccc} \text{ ​​}\lambda \text{​​}{\;}_{11}^{2}+\Psi_{1} & \cdots & \text{ ​​}\lambda \text{​​}{\;}_{11}\text{ ​​}\lambda \text{​​}{\;}_{1t}\\ \vdots & \ddots & \vdots \\ \text{ ​​}\lambda \text{​​}{\;}_{11}\text{ ​​}\lambda \text{​​}{\;}_{1t} & \cdots & \text{ ​​}\lambda \text{​​}{\;}_{11}^{2}+\Psi_{t} \end{array}\right]⊗\text{A}$, where $\Lambda =\left[\begin{array}{c} \text{ ​​}\lambda \text{​​}{\;}_{11}\\ \vdots \\ \text{ ​​}\lambda \text{​​}{\;}_{1t} \end{array}\right]$ and $\Psi =\left[\begin{array}{ccc} \Psi_{1} & 0 & 0\\ 0 & \ddots & 0\\ 0 & 0 & \Psi_{t} \end{array}\right]$ (Meyer, 2009). In addition, we calculated the genetic correlations (*r*_g_) among traits (i.e. heights at different TPs) (Supplementary Fig. S3), following the equation: $r_{g}=COV_{12}/\sqrt{\Sigma_{g_{1}}^{2}\Sigma_{g_{2}}^{2}}$, where COV_12_ was the genetic covariance between two traits; σ ^2^_g1_ and σ ^2^_g2_ were the genetic variances associated with each trait. The VCOV and genetic correlations were extracted from the ASReml-R output using the function met.corr2 from the AAfun set of additional functions for ASReml-R (https://github.com/yzhlinscau/AAfun/). Finally, broad-sense heritability (*h*^2^) was estimated based on an entry-mean for each TP, following the equation: *h*^2^=σ ^2^_g_/(σ ^2^_g+_σ ^2^_ε_), where σ ^2^_g_=*Vg* and σ ^2^_ε_=*Vε* were the genetic and residual variances (*V*), respectively. The SEs of heritability estimates were estimated using the ‘delta’ method (Venables and Ripley, 2000) using the pin R function (http://www.homepages.ed.ac.uk/iwhite/asreml/uop).

#### Smoothing and dimensionality reduction

We applied data smoothing followed by dimensionality reduction of genotype BLUPs across all TPs following the approach described by Kwak *et al.* (2016). First, we smoothed the data for each individual genotype using B-splines, choosing the number of basis splines to be used for all genotypes by minimizing the sum of squared errors from 10-fold cross-validation. Secondly, we applied functional principal component analysis (PCA) of the smoothed data to reduce their dimensionality. We used the funqtl (Kwak *et al.*, 2014) and fda (Ramsay et al., 2009, 2018) R packages for these analyses. The resulting coefficients of B-splines and functional principal components (PCs) were used for functional QTL mapping and genomic prediction (see below).

### Time point selection

We compared two approaches for selecting five or 10 TPs from the 26 TPs available for the 2017 data by sampling TPs in two different ways (Table 1). In the systematic (SY) approach, TPs were selected as equally spaced as possible (scenarios R_1_, R_2_, R_5_, and R_6_ in Table 1). This corresponds to a situation in which the image data are collected and processed throughout the growing season, without using previous knowledge about variability in different phenological stages. In contrast, in the growth stage (GS) approach, TPs were allocated preferentially to later stages (scenarios R_3,_ R_4_, R_7_, and R_8_ in Table 1) as phenotypic variation for height is known to increase over the course of the growing season (Holman *et al.*, 2016). This corresponds to a situation in which data collection and processing efforts are allocated based on previous knowledge and periodic inspection of the predominant phenological stage in the population.

**Table 1.** Systematic (SY) and phenological growth stage (GS) time point (TP) selection approaches for functional QTL mapping and genomic prediction in 197 wheat recombinant inbred lines (see the Materials and methods)

No. of TPs	Approach	Scenario	Days after sowing selected	Growth stage^*a*^			Multiple QTLs^*b*^			Genomic heritability^*c*^		
				TL	SE	HF	HK	ML	SL	PC1	BS (3, 4, 7)	BS (4, 5, 8)
5	SY	R_1_	23/70/128/181/228	3	1	1	5A 7A	2B	2B	0.23±0.02	0.20±0.02	0.20±0.02
	SY	R_2_	23/78/155/193/228	2	2	1	2B	2B	2B	0.23±0.02	0.22±0.02	0.20±0.02
	GS	R_3_	70/172/219/223/228	1	1	3	5A 7A	5A 7A	5A 7A	0.29±0.04	0.36±0.03	0.21±0.02
	GS	R_4_	181/214/219/223/228	0	1	4	2B 5A 7A	7A	7A	0.30±0.04	0.34±0.03	0.20±0.02
10	SY	R_5_	23/44/64/84/119/155/ 172/187/214/228	5	3	2	2B 5A 5B 7A	5A 7A	5A	0.26±0.03	0.30±0.04	0.21±0.02
	SY	R_6_	37/57/70/98/119/155/ 172/193/219/228	5	3	2	2B 5A 5B 7A	5A 7A	5A	0.26±0.04	0.29±0.04	0.24±0.03
	GS	R_7_	84/155/166/181/193/ 209/214/219/223/228	1	5	4	2B 5A 7A	5A	5A	0.28±0.04	0.32±0.03	0.24±0.03
	GS	R_8_	166/172/181/187/193/209/214/219/223/228	0	6	4	2B 5A 7A	5A	5A	0.28±0.04	0.34±0.04	0.21±0.02
26	–	R_9_	–	14	8	4	2B 5A 5B 7A	2B	5A 7A	0.28±0.04	0.32±0.02	0.21±0.04

^*a*^ Number of TPs selected in the tillering (TL), stem elongation (SE), and heading/flowering (HF) growth stages.

^*b*^ Chromosomes on which QTLs were detected at α=0.05 from the multiple functional QTL model based on multitrait (HKLOD), maximum (MLOD), and average (SLOD) score profiles.

^*c*^ Genomic heritability from the GBLUP model using dimension-reduced (first principal component, PC1) and the last two B-spline coefficients (BS) for five (BS 3–4), 10 (BS 4–5), and 26 (BS 7–8) TPs. Values are means ±SE estimated from 50 random cross-validations.

### Molecular markers

The 197 RILs were genotyped using the 35K Affymetrix Axiom^®^ HD wheat single nucleotide polymorphism (SNP) array (Allen *et al.*, 2017). Markers with significant segregation distortion and >20% missing data were removed, and a genetic linkage map was constructed as described previously by Allen *et al.* (2017). Briefly, the map included 9434 SNPs, covering a total of ~6632.3 cM of the wheat genome (21 linkage groups).

Markers with a low call rate (<90%) were removed, and the remaining missing SNP data were imputed using Beagle 4.1 (Browning and Browning, 2016) within the codeGeno function from the Synbreed R package (Wimmer *et al.*, 2012). Furthermore, markers with minor allele frequency (MAF) <0.01 and with redundant genotypes across all individuals were removed. The final molecular marker data consisted of 2330 SNPs, with the number of markers per chromosome ranging from 14 (chromosome 4D) to 223 (chromosome 5A; Supplementary Fig. S4).

### QTL mapping

QTL scanning was performed using both individual TP and functional mapping approaches because we were aiming to detect both transient and persistent QTLs. Thus, individual TP analysis was performed for each TP using the genotype BLUPs from the FA model utilizing the R/qtl package (Broman *et al.*, 2003). We performed both interval mapping (IM) and composite interval mapping (CIM) via Haley–Knott regression (Haley and Knott, 1992). For CIM, the number of marker covariates was set at five, with a window size of 10 cM. Logarithm of odds (LOD) thresholds for type I error rate (α=0.05) were obtained for each TP based on 1000 permutations.

Functional mapping was performed using the non-parametric approach suggested by Kwak *et al.* (2014, 2016) on the functional PCs (three, four, and five PCs for five, 10, and 26 TPs, respectively) using the funqtl R package. Single and multiple QTL analyses were carried out using the multitrait (HKLOD), maximum (MLOD), and average (SLOD) score approaches.

For the HKLOD approach, we used the following model:

$$\begin{array}{l} y=X\beta \;+\varepsilon \; \end{array}$$ (2)

where ***y*** is an *n*×*p* matrix of derived phenotypes (*n* genotypes×*p* functional PCs), ***X*** is an *n*×2 matrix of QTL genotype probabilities, **β** is a 2×*p* matrix of QTL effects, and **ε** is an *n*×*p* matrix of random residuals following a multivariate normal distribution. At each position λ (putative QTL location), a multivariate regression model with a single QTL was fitted. Thus, the log_10_ likelihood ratio of the HK model was $HKLOD=\frac{n}{2}log_{10}\left\{\frac{\left|RSS_{0}\right|}{\left|RSS\left(\lambda \right)\right|}\right\}$ where RSS was the matrix of sums of squares and cross-products of residuals, and |RSS| was its determinant; that is, |RSS_0_| was the matrix determinant for the null model with no QTL, and |RSS(λ)| was the matrix determinant for the model with a single QTL at position λ.

In contrast, the MLOD and SLOD approaches used the maximum and average LOD scores across TPs, respectively: MLOD(λ)=max_t_LOD(*t*,λ), and $SLOD\left(\lambda \right)=\frac{1}{T}\sum_{t=1}^{T}LOD\left(t,\lambda \right)$, where T is the number of TPs. LOD thresholds corresponding to α=0.05 were obtained for all three methods based on 1000 permutations using the scanoneM function of the funqtl package.

Finally, for multiple functional mapping (i.e. modelling several marker intervals simultaneously), we followed the methodology proposed by Kwak *et al.* (2014, 2016) in which the penalized LOD scores of Broman and Speed (2002) and Manichaikul *et al.* (2009) were used to estimate the HKLOD, MLOD, and SLOD scores. Initially, we selected the main effect, heavy chain, and light chain penalties obtained from 1000 permutations (α=0.05) using the scantwoF function. Then, we used the stepwise model search algorithm (Broman and Speed, 2002) to scan the genome using forward selection of a model with up to six fixed QTLs, followed by backward elimination to the null model using the stepwiseqtlM function.

### Power simulations

We performed data perturbation simulations (Yu *et al.*, 2006) to assess the statistical power of detecting persistent and transient QTLs using individual TP and functional mapping analyses. For each of 1000 iterations of the simulations, we randomly chose an SNP from the genotypic data set and assigned constant phenotypic effects –α and α (Falconer and Mackay, 1995) to alternative homozygous genotypes for that SNP (i.e. there were no heterozygotes in the data). These effects were then added to BLUPs for all TPs (persistent QTLs) or for a single, randomly selected TP (transient QTLs), and the resulting values were used as phenotypes for QTL mapping (i.e. as described above). We varied the additive effects to achieve different proportions of variance explained (PVE) using the relationship $PVE=\frac{1}{1+\frac{1}{k^{2}p\left(1-p\right)}}$ (Yu *et al.*, 2006; Slavov *et al.*, 2014), where *p* is the estimated frequency of an arbitrarily chosen allele and *k* is the simulated additive effect divided by the SD of the BLUPs for the respective TP. Finally, statistical power was estimated as the proportion of iterations in which the simulated additive effects were detected as QTLs at α=0.05 based on the permutation thresholds used in our analyses of empirical data (see above).

### Genomic prediction

The phenotypic traits used for prediction were the (i) individual TP BLUPs; (ii) the last two B-spline coefficients; and (iii) the first functional PCs from analyses of five, 10, and 26 TPs, including different TP selection scenarios (R_1_–R_8_, Table 1).

We used the additive genomic best linear unbiased prediction (GBLUP) model:

$$\begin{array}{l} \hat{y}=X\beta \;+Za+\varepsilon \; \end{array}$$ (3)

where $\hat{y}$ was a vector of phenotypic values, **β** was the vector of fixed effects, ***a*** was the vector of random additive genetic effects of the SNP markers, and **ε** was a vector of random residuals. The incidence matrices for **β** and ***a*** were ***X*** and ***Z***, respectively. The distributions of random effects were assumed to be ***a***~*N*(0,σ ^2^_a_***G***_**a**_) and ***ε***~*N*(0,σ ^2^_ε_***I***), where ***I*** was the identity matrix and ***G***_**a**_ was the additive genomic relationship matrix calculated using the first formula proposed by VanRaden (2008).

We also integrated QTL mapping information into the GBLUP model (Equation 3). This model is expected to be advantageous assuming that height is controlled by relatively few genes of large effect and many genes of smaller effect (Sarinelli *et al.*, 2019). Initially, we performed a single HKLOD functional mapping scan to identify significant cofactor SNPs at α=0.05 based on the 1000 permutations in each training set (148 individuals). Then, these cofactor SNPs, if any were detected, were included as fixed-effect covariates into the GBLUP model. For this analysis, we only used the systematic R_1_ and R_5_ scenarios, as well as the full set of 26 TPs (R_9_; Table 1).

Predictive ability (*r*) was calculated as the Pearson correlation between adjusted values and genomic estimated breeding values in 50 replications from independent validation scenarios (Albrecht *et al.*, 2014), randomly sampling 75% of the genotypes (*n*=148) to form a training set, while the remaining 25% (*n*=49) were used as a validation set. We applied Fisher’s Z transformation of the predictive abilities and compared them among scenarios using Scott–Knott’s test (Scott and Knott, 1974) at α=0.05. All prediction analyses were performed using the Bayesian Generalized Linear Regression (BGLR) R package (Perez and de los Campos, 2014), using 60 000 Markov Chain Monte Carlo (MCMC) iterations, with 15 000 iterations for burn-in, and keeping only one from every five consecutive iterations to minimize auto-correlation. In addition to predicted phenotypes, we extracted genomic estimates of the additive genetic (σ ^2^_a_=*V*_g_*a*) and residual (σ ^2^_ε_=*V*_g_*ε*) variances, enabling the calculation of genomic heritability as *h*^2^_g_=σ ^2^_a_/(σ ^2^_a_+σ ^2^_ε_).

## Results

### Genetic variances and temporal covariances in height increased over time

**Fig. 1.**
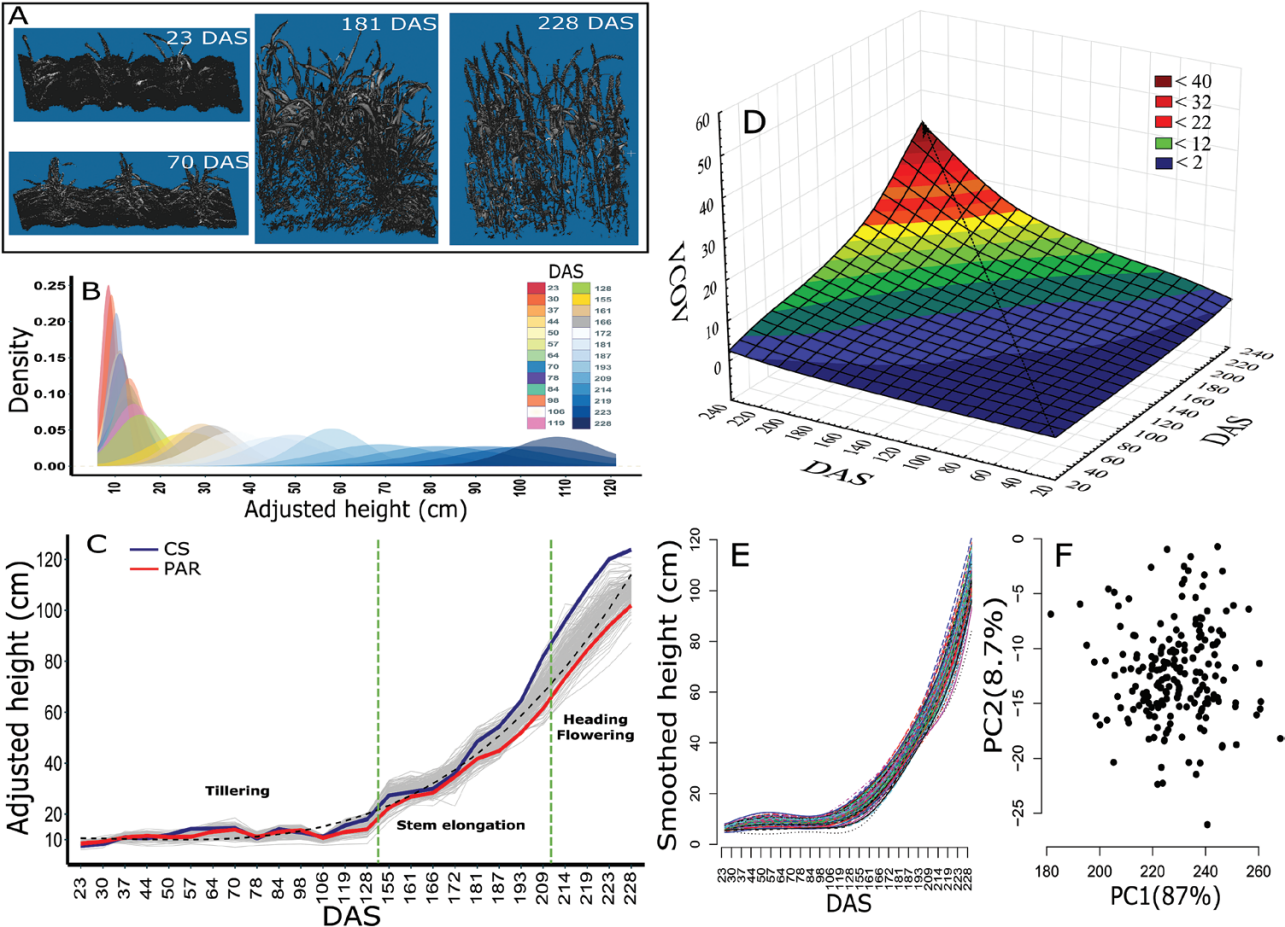
Temporal pattern of canopy height growth in a wheat recombinant mapping population measured from a robotic Field Scanalyzer in 2017. (A) 3D canopy images at 23, 70, 181, and 228 days after sowing (DAS) from one field plot. (B) Density distributions of the adjusted phenotypic values (best linear unbiased predictions, BLUPs) within time points (TPs) over the course of the growing season. (C) Adjusted height values of 197 recombinant inbred line genotypes, and both parents Chinese Spring (CS) and Paragon (PAR) from 26 TPs. The dotted line shows the estimated cubic spline curve across all genotypes. The mean broad-sense heritability across all TPs was 0.68. The growth stages delineated by dashed lines are tillering (23–128 TP), stem elongation (155–209 TP), and heading/flowering (214–228 TP). (D) Temporal genetic variance–covariance structure (VCOV) surface plot. (E) B-spline smoothed and (F) dimension-reduced [first (PC1) and second (PC2) functional principal components] height phenotypes based on 26 TPs. Percentages in parentheses represent the variance explained by each of the two PCs. (This figure is available in colour at *JXB* online.)

We generated 3D height images (Fig. 1A) for each plot under the robotic Field Scanalyzer over the entire crop growth cycle. The mean broad-sense heritability across all TPs was 0.68 for the 2017 data. As expected, both variances and covariances among TPs increased over the course of the growing season (Fig. 1B–D). In the 2016 data set, the mean broad-sense heritability was 0.57 (Supplementary Fig. S5A, B). However, due to the low quality of the 2016 data set, particularly during the late stages of the growing season (see the Materials and methods), most downstream analyses were limited to data from 2017.

To enable comparisons among different TP selection strategies (Table 1), we used B-spline smoothing followed by functional PCA of the adjusted values for each scenario. The resulting temporal pattern was consistent among scenarios, initially following an accelerating and then a linear trend, with distinct, genotype-specific trajectories, including for the two RIL parents (i.e. significant genotype×TP interaction effects, *P*<0.01) (Fig. 1C, E; Supplementary Fig. S6A, B). The proportion of variance explained by the first two PCs was high (93–96%) and increased slightly with the number of TPs analysed (Fig. 1F; Supplementary Fig. S6C, D).

### Individual time point analyses detected multiple QTLs across all developmental stages

**Fig. 2.**
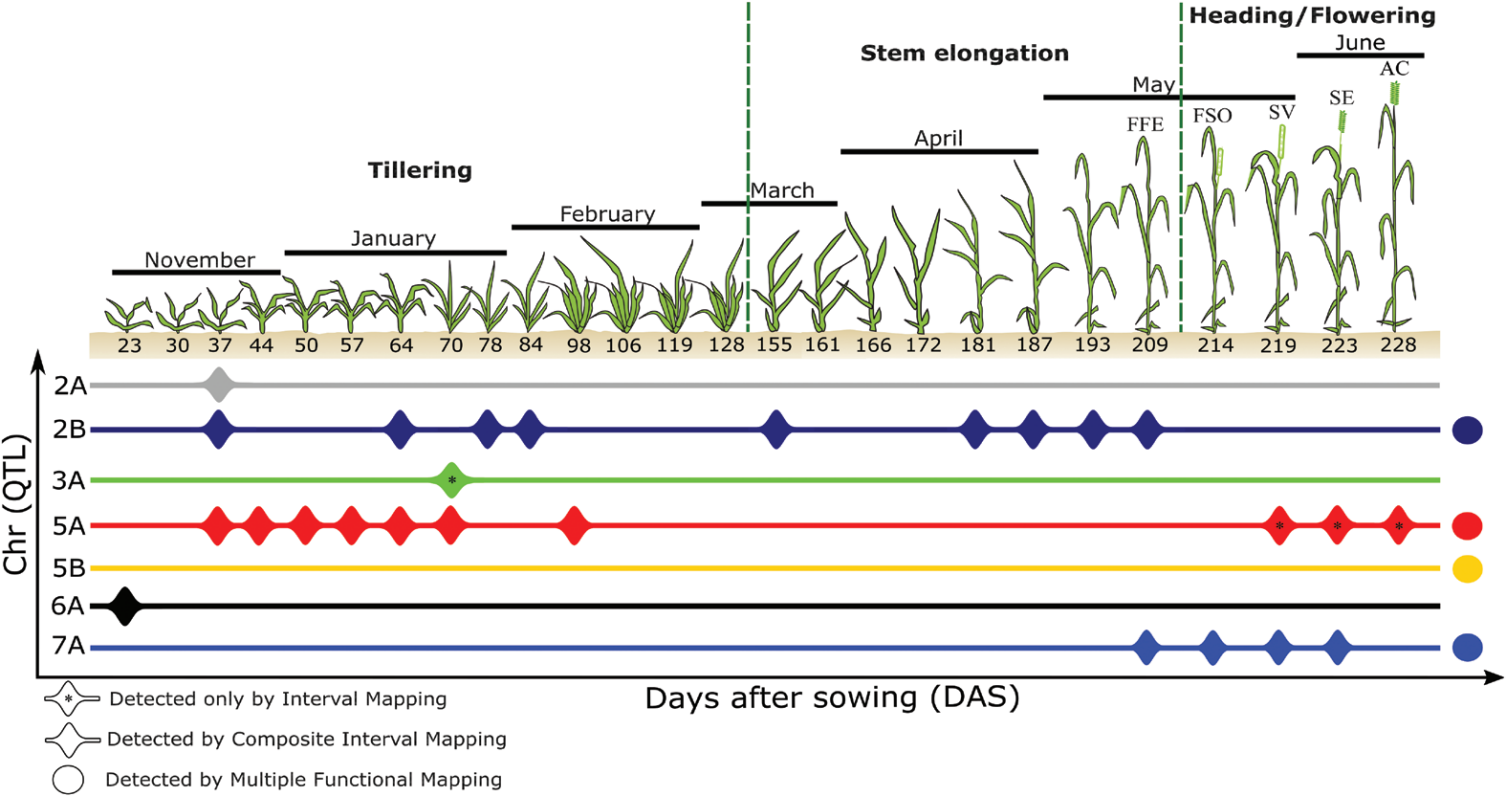
Summary of individual time point (TP) and functional QTL mapping results in 2017. Growth stages are defined as in Fig. 1. Chr, chromosome on which a QTL was detected; FFE, flag leaf fully emerged; FSO, flag leaf sheath opening; SV, first spikelet visible; SE, spike 100% emerged; AC, anthesis completed. (This figure is available in colour at *JXB* online.)

From the IM and CIM scans of the 2017 data (i.e. analysing data from each TP separately), we detected a total of six QTLs (Fig. 2; Supplementary Figs S7, S8A). Three of these (i.e. on chromosomes 2A, 3A, and 6A) were associated with height only during the tillering growth stage, whereas one (on chromosome 7A) was detected exclusively during late stages. In contrast, the QTL on chromosome 2B was detected in both the tillering and stem elongation (mid) stages, whereas the QTL on chromosome 5A was, surprisingly, detected in the tillering and heading stages, but not the intervening stem elongation stage.

Based on the 2016 data, we detected one QTL on chromosome 2B across multiple TPs, whereas another QTL on chromosome 6A was detected in a single TP, in a different growth stage from 2017 (Supplementary Figs S8B, S9A). Furthermore, analyses of the manual plant height data from 2016 resulted in the detection of two additional late-stage QTLs on chromosomes 1A and 2D (Supplementary Fig. S9B–E).

### Additional QTLs could be detected using functional mapping

**Fig. 3.**
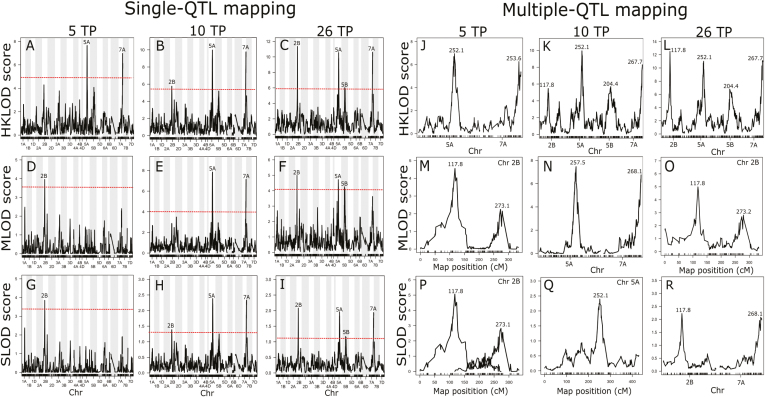
Functional QTL mapping analysis for five, 10, and 26 time points (TPs) in 2017. (A–I) Single and (J–R) multiple QTL approach based on multitrait (HKLOD), maximum (MLOD), and average (SLOD) profiles. The 5 and 10 TP results correspond to the systematic R_1_ and R_5_ scenarios (see Table 1). Dashed lines indicate the α=0.05 thresholds. Numbers above peaks in the multiple QTL mapping plots represent the positions of the loci on the respective chromosomes (Chr). (This figure is available in colour at *JXB* online.)

Using single functional mapping at five, 10, and 26 TPs based on the multivariate LOD test statistic (HKLOD score), we identified two, three, and four QTLs, respectively (Fig. 3A–C). Results from the MLOD and SLOD approaches were largely consistent with this (Fig. 3D–I) as were results from multiple functional mapping analyses (Fig. 3J–R). Most importantly, we detected an additional QTL on chromosome 5B that was consistently identified based on single and multiple functional QTL mapping (Figs 2; 3C, F, I, K, L), but not in scans of individual TP data (Fig. 2; Supplementary Fig. S7). As with individual TP approaches, analyses of the 2016 data using single QTL mapping only resulted in the detection of the QTL on chromosome 2B (Supplementary Fig. S10A–I).

**Fig. 4.**
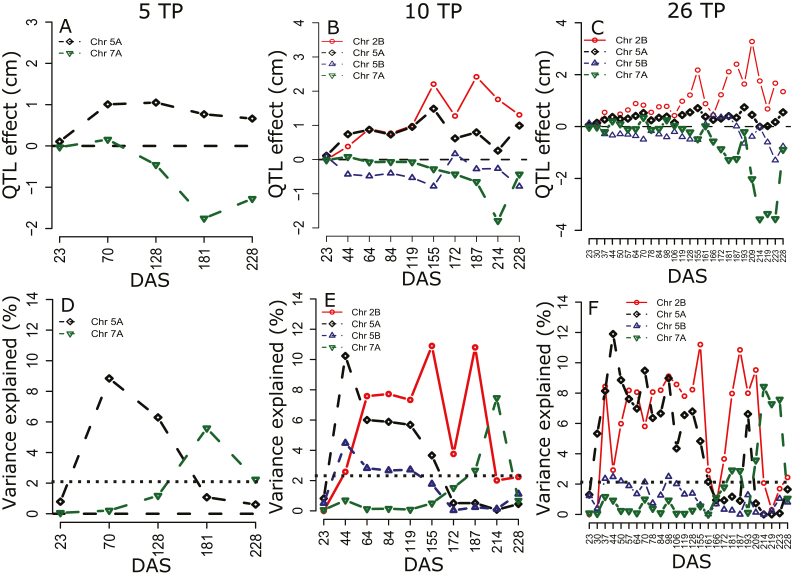
Estimated QTL effect sizes for analyses of five, 10, and 26 time points (TPs) in 2017. (A–C) Estimated absolute QTL effects and (D–F) percentage of phenotypic variance explained (PVE%), by the QTLs in the multiple functional mapping based on the multivariate LOD test statistic (HKLOD score). The black dotted line in (D–F) indicates α=0.01. The 5 and 10 TP results correspond to the systematic R_1_ and R_5_ scenarios (see Table 1). DAS, days after sowing. Signs of QTL effects indicate allele substitution effects: Chinese Spring was the donor of height-increasing alleles for the QTLs on chromosomes 2B and 5B, whereas Paragon contributed height-increasing alleles for the QTLs on chromosomes 5A and 7A. (This figure is available in colour at *JXB* online.)

**Fig. 5.**
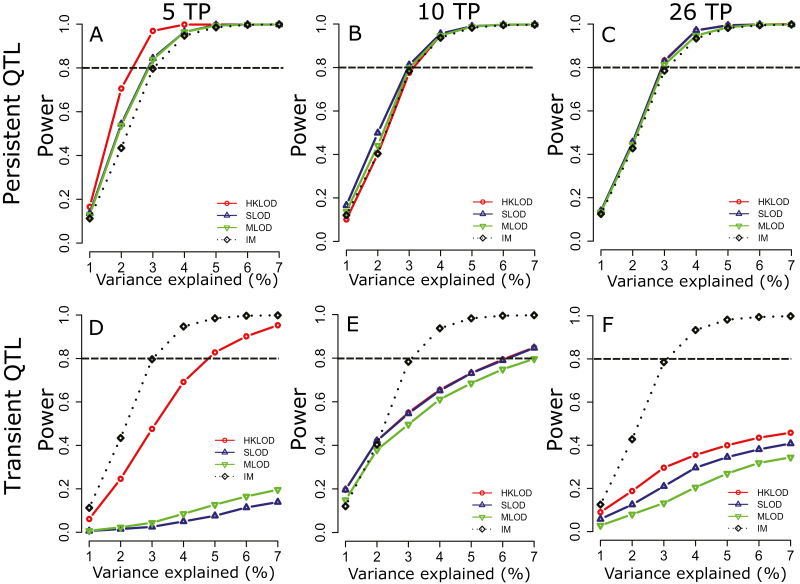
Statistical power for analyses of five, 10, and 26 time points (TPs) in 2017. Power for persistent (A–C) and transient (D–F) QTLs was calculated at α=0.05 based on data perturbation simulations, and functional mapping multitrait (HKLOD), maximum (MLOD), and average (SLOD) profiles compared with interval mapping (IM) analyses. The 5 and 10 TP results correspond to the systematic R_1_ and R_5_ scenarios (see Table 1). (This figure is available in colour at *JXB* online.)

As expected under the presumed genetic model of highly complex temporal dynamics of height growth, both absolute effect sizes and proportions of PVEs varied substantially across the growing season, with clear examples of early (5A, 5B), late (7A), or persistent (2B) QTLs (Fig. 4A–F). Furthermore, despite some variability in the relative performances of HKLOD, MLOD, and SLOD models, power simulations demonstrated a slight advantage of functional mapping approaches (i.e. versus conventional individual TP analyses) for the detection of persistent QTLs (Fig. 5A–C). In contrast, individual TP scans had substantially greater power for detecting transient QTLs (Fig. 5D–F).

### Individual time point genomic heritability and predictive ability were stable across the growing season

**Fig. 6.**
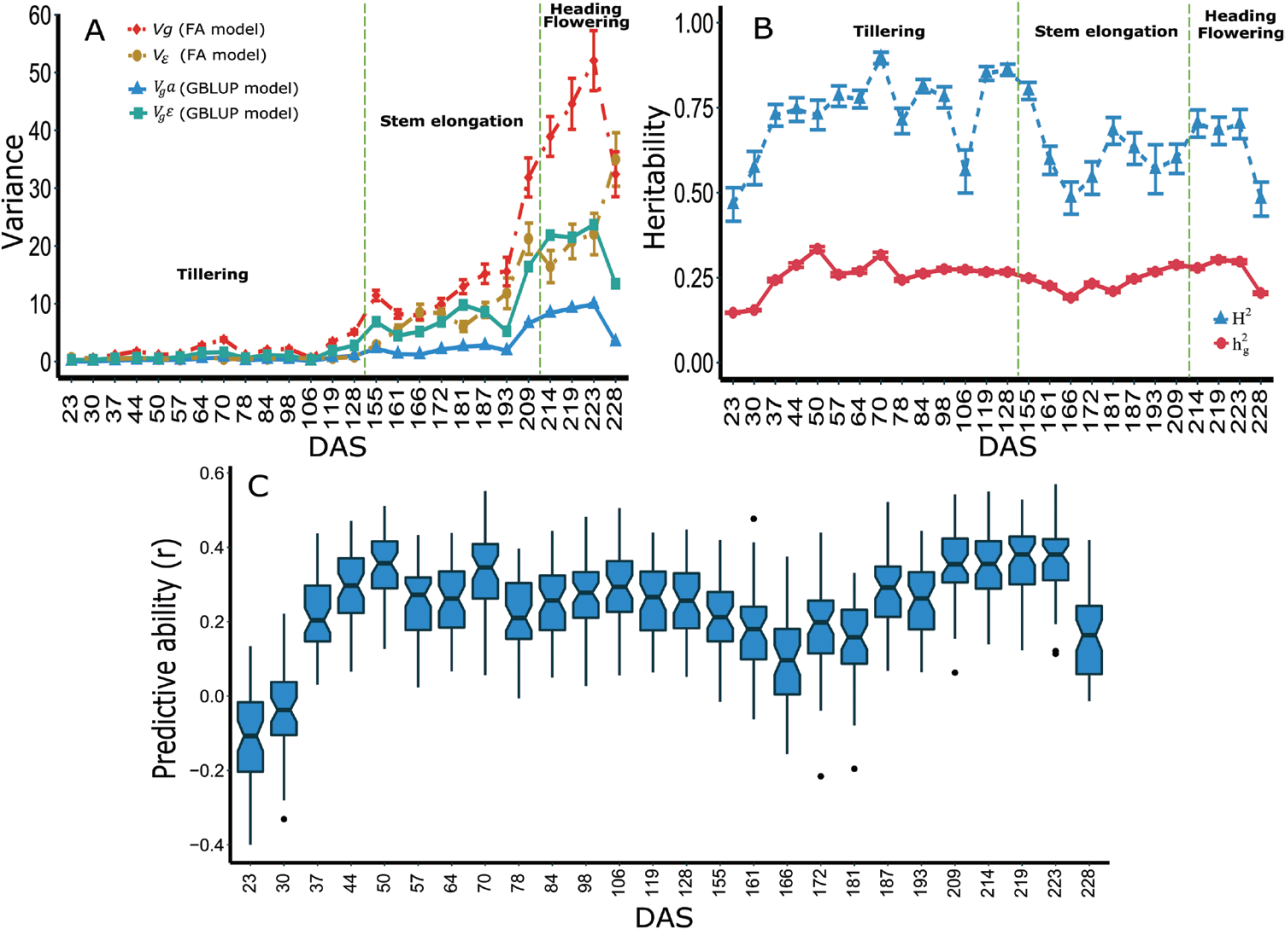
Genomic prediction of canopy height across 26 individual time points (TPs). (A) Variance estimates for each TP from the phenotypic (factor analytic, FA) and genomic (genomic best linear unbiased predictor, GBLUP) model. Growth stages are defined as in Fig. 1. (B) Broad-sense (*H*^2^) and genomic (*h*^2^_g_) heritability based on the phenotypic FA and GBLUP models, respectively. Error bars represent SEs. (C) Predictive ability (*r*) based on the GBLUP model. DAS, days after sowing. (This figure is available in colour at *JXB* online.)

Genetic and residual variance components from the GBLUP model generally followed the same temporal patterns as those from the FA model, with increasing values after the tillering growth stage (Fig. 6A). Consequently, the time trend of genomic heritability resembled that of broad-sense heritability, stabilizing at ~0.25 after the first two TPs (Fig. 6B). As expected, genomic predictive ability followed a similar pattern, fluctuating at ~0.30 and decreasing slightly at the last TP, presumably because of lodging, which affected data quality for some genotypes (Figs 1C, 6C). As expected from the lower quality of the 2016 data set (see the Materials and methods), we observed very low individual TP predictive abilities (ranging from 0 to 0.1; results not shown).

### Smoothing height phenotypes reduces noise and increases predictive ability

**Fig. 7.**
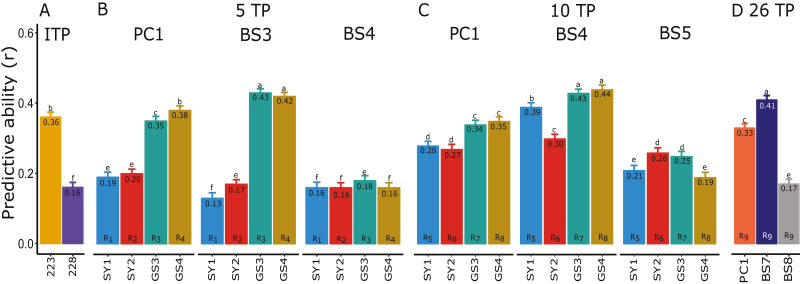
Functional genomic prediction and assessment of time point (TP) selection strategies. (A) Predictive abilities for the ‘best’ (i.e. having the highest predictive ability, DAS=223) and last (DAS=228) individual TPs. (B–D) Predictive abilities for dimension-reduced phenotypes (first principal component, PC1), and the last two B-spline basis coefficients (BS) for five, 10, and 26 TPs. TP selection strategies (B, C) followed a systematic approach (SY) or were informed by phenological growth stages (GS) as shown in Table 1. Numbers inside plots and error bars represent the mean and SE from 50 random cross-validations. Letters above bars indicate significant differences at α=0.05 across all methods. DAS, days after sowing. (This figure is available in colour at *JXB* online.)

**Fig. 8.**
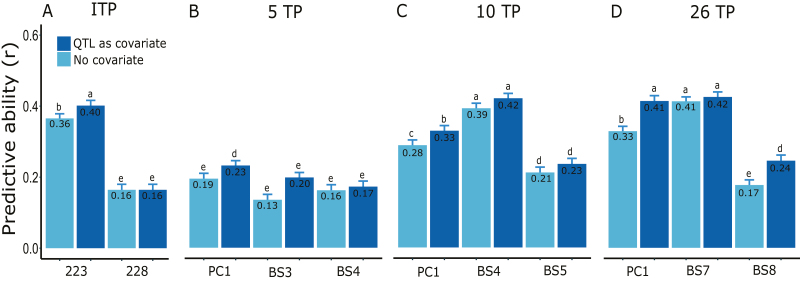
Genomic prediction including functional QTLs as fixed-effect covariates. (A–D) Predictive ability with and without QTLs detected in the training population as fixed-effect covariates in the GBLUP model. (A) Results for the ‘best’ (i.e. having the highest predictive ability, DAS=223) and last (DAS=228) individual time points (TPs). (B–D) Results for dimension-reduced (first principal component, PC1), and the last two B-spline coefficients (BS) for five, 10, and 26 TPs. The 5 and 10 TP results are for the systematic R_1_ and R_5_ scenarios (see Table 1). Numbers inside plots and error bars represent the mean and SE from 50 random cross-validations. Letters above bars indicate significant differences at α=0.05 across all methods. DAS, days after sowing. (This figure is available in colour at *JXB* online.)

The predictive ability for B-spline coefficients of phenotypes that had been smoothed was generally higher than that for individual TP or PC data (Fig. 7). Based on our evaluation of different TP selection strategies, there was a clear advantage (i.e. from ~10% to >3-fold) of using phenological information compared with systematically interspersed sampling, particularly when a small number of TPs were evaluated (Fig. 7B; Table 1). Interestingly, increasing the number of TPs analysed beyond five did not improve predictive abilities (Fig. 7B–D). Finally, including functional QTLs detected in the training population as fixed-effect covariates in the GBLUP model tended to increase predictive ability slightly, particularly for PC phenotypes (Fig. 8).

## Discussion

### Genetic variances and temporal covariances in height increased over time

Previous longitudinal studies of plant height suggested that both genetic and environmental variation tend to increase during the growing season, presumably because of the cumulative effects of large numbers of interacting genetic loci and environmental cues (Wang *et al.*, 2010; Wu *et al.*, 2010; Holman *et al.*, 2016; Davey *et al.*, 2017). Consistent with this expectation, we observed higher genetic variability in later developmental stages and increasing temporal genetic covariance, but also substantially higher residual (i.e. presumably, to a large extent, environmental) variation during the second half of the growing season (Figs 1C–E; 6A). Consequently, both broad-sense heritabilities increased steeply during the early part of the tillering phase and then fluctuated considerably during the stem elongation and flowering phases (Fig. 6B). Similar patterns of highly variable heritability during crop growth have been reported across a range of complex traits by high-throughput phenotyping studies in wheat (Madec *et al.*, 2017), rice (Campbell *et al.*, 2017), barley (Chen *et al.*, 2014), and *Setaria* (Feldman *et al.*, 2017). Thus, the distribution of sampled TPs across developmental and/or phenological stages is an important practical consideration.

### Individual time point analyses detected multiple QTLs across all developmental stages

All of the QTLs detected in our study (Figs 2–4) are on chromosomes for which QTLs have been reported previously (Wu *et al.*, 2010; Griffiths *et al.*, 2012; Bentley *et al.*, 2014; Zanke *et al.*, 2014; Gao *et al.*, 2015; Wurschum *et al.*, 2015; Tian *et al.*, 2017; Guan *et al.*, 2018). For example, Griffiths *et al.* (2012) detected meta-QTLs on chromosomes 2A, 2B, 3A, 5A, and 6A based on analyses of four mapping populations. Similarly, Gao *et al.* (2015) identified QTLs on chromosomes 2B, 5A, and 7A in a set of RILs from a cross between Zhou8425B and CS (i.e. one of the parents used in our study). Thus, it seems plausible that some of the QTLs detected in our analyses are caused by variation in well-known *Rht* dwarfing genes (Ellis *et al.*, 2005; Wu *et al.*, 2010). However, we did not detect any strong signals on homoeologous chromosome group 4, presumably because the well-characterized *Rht-B1* and *Rht-D1* loci (Wurschum *et al.*, 2017, 2018) do not segregate in the cross we studied (Gao *et al.*, 2015; Kowalski *et al.*, 2016).

Interestingly, our individual TP analyses resulted in the detection of putatively transient QTLs, some of which were not detectable using functional mapping approaches (Fig. 2). This finding is consistent with a number of recent studies that have reported temporally dynamic QTL expression in wheat (Zhang *et al.*, 2017; Camargo *et al.*, 2018) and other crops (Busemeyer *et al.*, 2013; Al-Tamimi *et al.*, 2016; Campbell *et al.*, 2017; Feldman *et al.*, 2017; Muraya *et al.*, 2017; Ward *et al.*, 2019). However, because transient QTLs are inherently more difficult to detect (i.e. due to their typically smaller effect sizes and narrow windows of expression) and are more likely to be false positives, their overall contribution to the genetic architecture of complex traits is currently unclear. Addressing this question would require using much larger mapping populations and possibly analyses of more TPs. Technological advances should make considerable upscaling possible in the near future (Roitsch *et al.*, 2019).

### Functional analyses increased QTL detection power and genomic predictive ability

Our functional mapping analyses resulted in the detection of an additional QTL on chromosome 5B (Figs 2–4), which appeared to affect plant height growth primarily during the tillering stage (Fig. 4E, F). A QTL has previously been reported on the same chromosome, also with effects predominantly in early phenological stages (Wang *et al.*, 2010; Wu *et al.*, 2010).

One of the motivating arguments for the development of functional mapping approaches was the premise that these approaches would have greater statistical power than conventional QTL mapping of data from individual TPs (Ma *et al.*, 2002; Wu *et al.*, 2004; Wu and Lin, 2006). Both our empirical results (Fig. 2) and power simulations (Fig. 5A–C) were consistent with this expectation, but only for persistent QTLs. In contrast, individual TP analyses resulted in the detection of higher numbers of transient or stage-specific QTLs (Fig. 2), which was also consistent with our power simulations for transient QTLs (Fig. 5D–F). Therefore, combining both individual TP and functional mapping approaches may be preferable as these approaches are able to capture different types of QTLs (Bac-Molenaar *et al.*, 2015; Campbell *et al.*, 2017; Muraya *et al.*, 2017; Camargo *et al.*, 2018).

The HKLOD method often outperformed the MLOD and SLOD approaches in analyses of both empirical (Fig. 3) and simulation data (Fig. 5). However, this pattern was far from consistent, in agreement with previous findings about the contrasting characteristics of these approaches Kwak *et al.* (2014, 2016). Given their relatively low computational cost, we therefore recommend using all three functional mapping methods.

Unlike the highly variable conventional estimates of heritability (i.e. based exclusively on phenotypic data, Fig. 6A), genomic heritability and predictive ability were relatively stable throughout the growing season, particularly after the early part of the tillering stage (Fig. 6B, C). Thus, genomic prediction based on data from a single, appropriately chosen TP would be reasonably accurate. However, we identified three factors that can further increase predictive ability. First, predictions of smoothed height phenotypes (i.e. multiple TPs) were >20% more accurate than those of phenotypes from individual TPs (Fig. 7), presumably as a result of the reduction of measurement error (Kwak *et al.*, 2016). Consistent with this explanation, genomic heritabilities of the smoothed data were on average 7% higher than those of individual TPs (Table 1 versus Fig. 6B). Second, we observed a slight increase in predictive ability when including significant QTLs as fixed-effect covariates in the GBLUP model (Fig. 8). This is consistent with several previous empirical and simulation studies (Bernardo, 2014; Arruda *et al.*, 2016; Spindel *et al.*, 2016; Bian and Holland, 2017; McElroy *et al.*, 2018; Sarinelli *et al.*, 2019). Finally, as expected based on general knowledge about wheat growth and development (Fischer, 2011; Fischer and Rebetzke, 2018; Dreccer *et al.*, 2019), predictive abilities were up to three times higher when TPs were chosen based on phenological growth stages compared with a systematically interspersed selection of TPs throughout the growing season (Fig. 7). Furthermore, as in previous studies (Camargo *et al.*, 2016; Rutkoski *et al.*, 2016), our findings emphasized the importance of sampling during the reproductive crop stage (i.e. heading and flowering), presumably because of the higher genetic variance captured during that stage (Table 1; Figs 6, 7).

### Recommendations

The crux of our findings is the existence of a trade-off between the extraction of data for large numbers of TPs (i.e. to detect as many transient or stage-specific QTLs as possible) and the additional computational cost (mostly image processing but also downstream analyses) that may not necessarily translate into improved genomic prediction or more complete understanding of trait architecture. Based on our results, we formulated the following two practical recommendations, which are naturally only applicable in situations similar to our study. First, when the emphasis is on dissecting trait architecture, we recommend using both individual TP and functional mapping approaches to maximize the overall number and types of QTLs detected. Secondly, for more applied purposes (e.g. in breeding), analyses of spline-smoothed phenotypes selected based on phenological information may be sufficient to maximize genomic predictive ability. Further slight improvements may be achieved through the inclusion of QTLs as fixed-effect covariates, though it is not currently clear if the additional complexity (and consequently delay) associated with QTL detection is justified.

## Supplementary data

Supplementary data are available at *JXB* online.

Fig. S1. Illustration of steps for processing of point clouds.

Fig. S2. Manual ground height versus height from the 3D point cloud.

Fig. S3. Heatmap of genetic correlations of height between 26 TPs in 2017.

Fig. S4. Genetic map consisting of 2330 SNPs.

Fig. S5. Temporal pattern of canopy height growth in 2016.

Fig. S6. Smoothed and dimension-reduced height in 2017.

Fig. S7. Interval and composite QTL mapping at 26 TPs in 2017.

Fig. S8. Signed LOD scores for each time point in 2017 and 2016.

Fig. S9. Summary of individual TP mapping results in 2016.

Fig. S10. Functional QTL mapping analysis for five, 10, and 22 TPs in 2016.

Table S1. Likelihood ratio and Wald tests at 26 TPs in 2017.

erz545_suppl_Supplementary_Figures_S1-S10_and_Table_S1

## Data Availability

The phenotypic and genotypic data, as well as the R scripts used for all analyses in this study, can be found at Mendeley (Lyra, 2019; https://data.mendeley.com/datasets/pkxpkw6j43/2).

## Abbreviations

BLUP: best linear unbiased prediction
DAS: days after sowing
GBLUP: genomic best linear unbiased prediction
HKLOD: multitrait method score
HTPP: high-throughput plant phenotyping
MLOD: maximum score
PC: principal component
QTL: quantitative trait locus
SLOD: average score
SNP: single nucleotide polymorphism
TP: time point.
