# Supplementary material for: Functional QTL mapping and genomic prediction of canopy height in wheat measured using a robotic field phenotyping platform
Source: J Exp Bot. 2020 Feb 25;71(6):1885–98. doi: 10.1093/jxb/erz545 (PMC7094083; doi:10.1093/jxb/erz545)
Supplement: erz545_suppl_Supplementary_Figures_S1-S10_and_Table_S1 [file erz545_suppl_supplementary_figures_s1-s10_and_table_s1.pdf]

SUPPLEMENTARY DATA

3D point clouds processing for canopy height extraction

Data were collected using the twin laser scanner of the Field Scanalyzer platform (Virlet *et al.*, 2017). Lasers are class 3B with an opening angle of 15°. They display a field of view of ~0.5 m width and 0.5 m depth of view. The scan length depends on the settings. In this study, both lasers were collecting scans of 3 m length with an overlapped region of 1.5 m.

The measurements were done on an office PC running Windows OS with 4-core Intel, 3.2 Ghz per CPU and 8 GB of memory. The algorithm was developed using python libraries (e.g. point cloud library, PCL, http://pointclouds.org/).

The steps to measure canopy height were as follows (see Supplementary Fig. S1):

1. First, we merged the two-point clouds collected from the twin laser scanner to complete surface reconstruction. Then, a registration technique known as Iterative Closest Point (ICP) (Romero and Felix, 2008) was used to minimise the distance between the two clouds. The ICP iteratively revises the transformation composed of rotation, scale and translation used to minimize the distance between the points of the two clouds. The point correspondences were extracted considering the closest point in the other view.
2. For each RIL or parent, the region of interest (RoI) was identified by cropping the area in all three axes (2015-2016: 1 row per line; 2016-2017: 3 rows per lines).
3. The RoI was then divided into sub regions, sRoIn (n = 3 for 2015-2016 and n = 9 for 2016-2017)
4. For each sRoIn the following steps were executed:
    a. First the point cloud with the highest z-value (mm) was identified as the maximum height (maxH) value for that individual sRoIn,
    b. Then, 10% of the maxH was calculated and subtracted from maxH,
    c. Finally, the average value of all points within the interval between maxH and 10%maxH was calculated as the Top_height_sRoIn,
    d. Since soil varies from one sRoIn to another, the soil level was calculated automatically for each sRoIn at the beginning of the season and used thereafter throughout the whole season:
        i. For each sRoIn, the point cloud density histogram of the z axis was plotted,
        ii. It was observed that soil displays a higher point cloud density. Thus, the highest peak of the histogram represented the location of the mode of the points and the soil level,
        iii. The soil level was then subtracted for each of the sRoI to obtain the canopy height: Canopy_height_sRoIn.
5. The final canopy height was computed by averaging all the Canopy_height_sRoIn values.

References

Romero L, Felix CA. 2008. Robust iterative closest point algorithm with augmented features advances in artificial intelligence. Springer, v. 5317, pp. 605-614.

Virlet N, Sabermanesh K, Sadeghi-Tehran P, Hawkesford MJ. 2017. Field Scanalyzer: An automated robotic field phenotyping platform for detailed crop monitoring. Functional Plant Biology 44, 143-153.

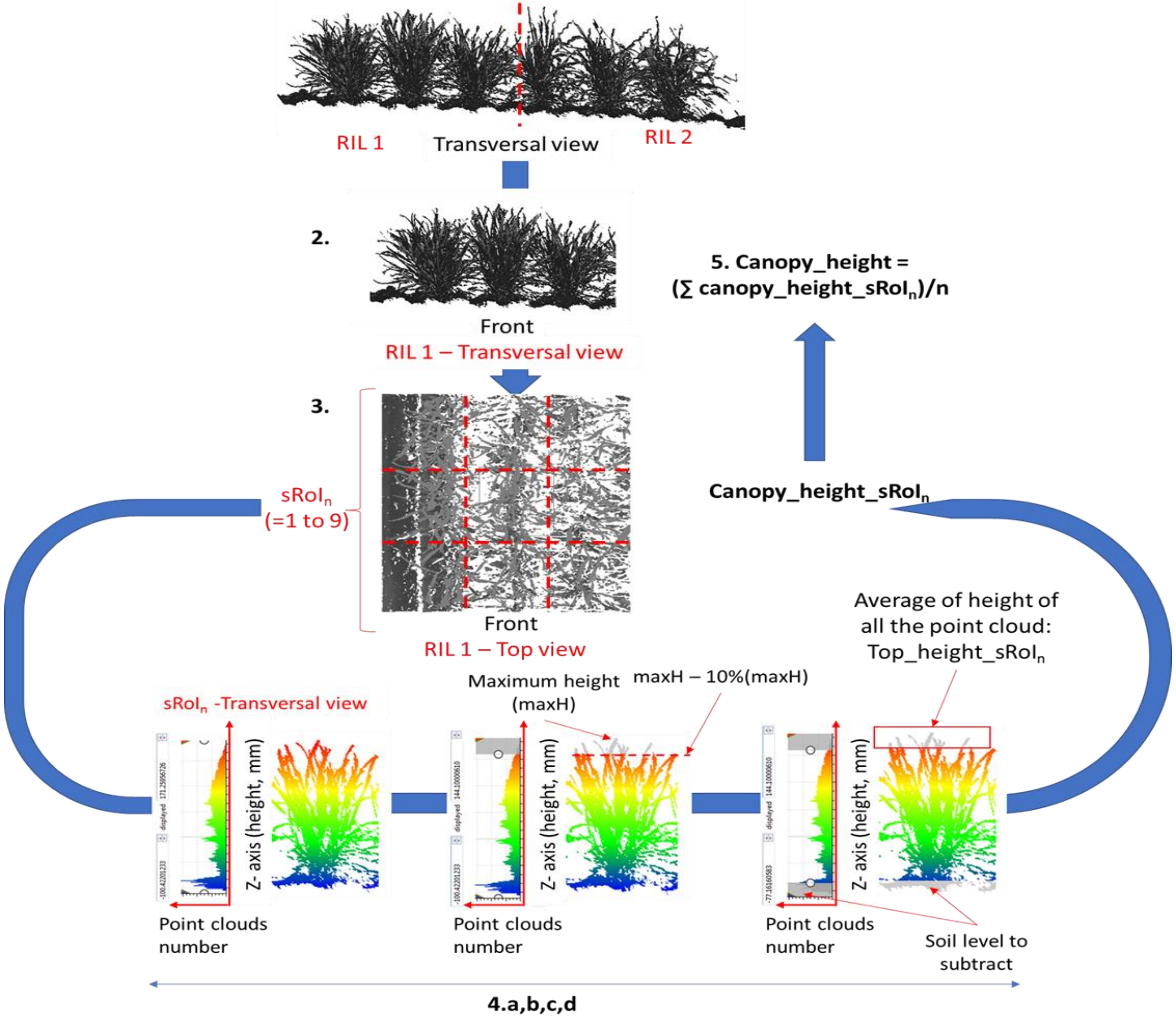

Supplementary Fig. S1. Illustration of the steps of the 3D point clouds processing. For detailed description, see "3D point clouds processing for canopy height extraction", above. The numbers (steps 2 to 5) corresponds to the text in that section.

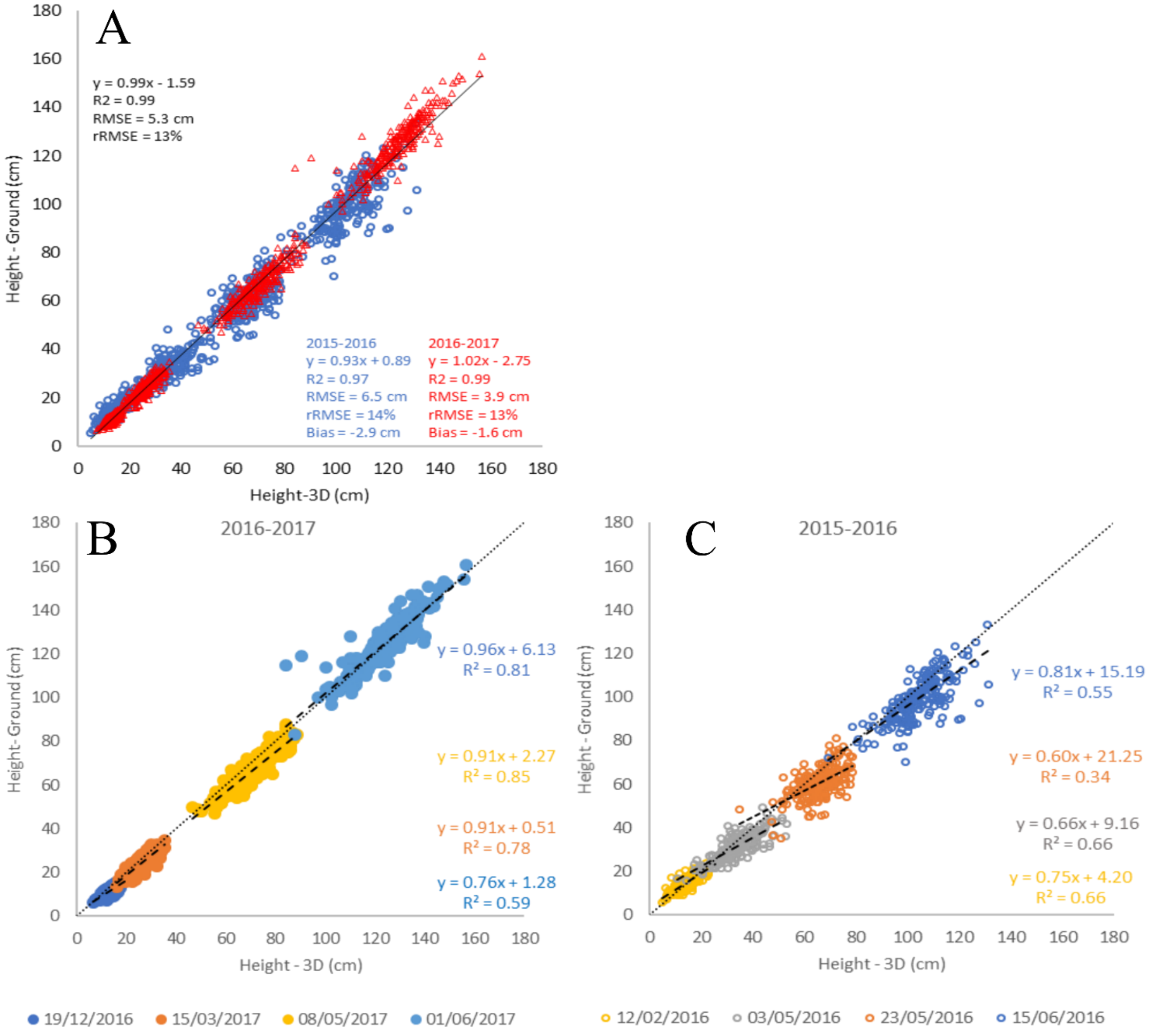

Supplementary Fig. S2. Manual height measurement vs height from the 3D point-cloud for (A) both 2016 and 2017, (B) 2017 and (C) 2016 data sets. R^2: coefficient of determination; RMSE: root-mean-square error.

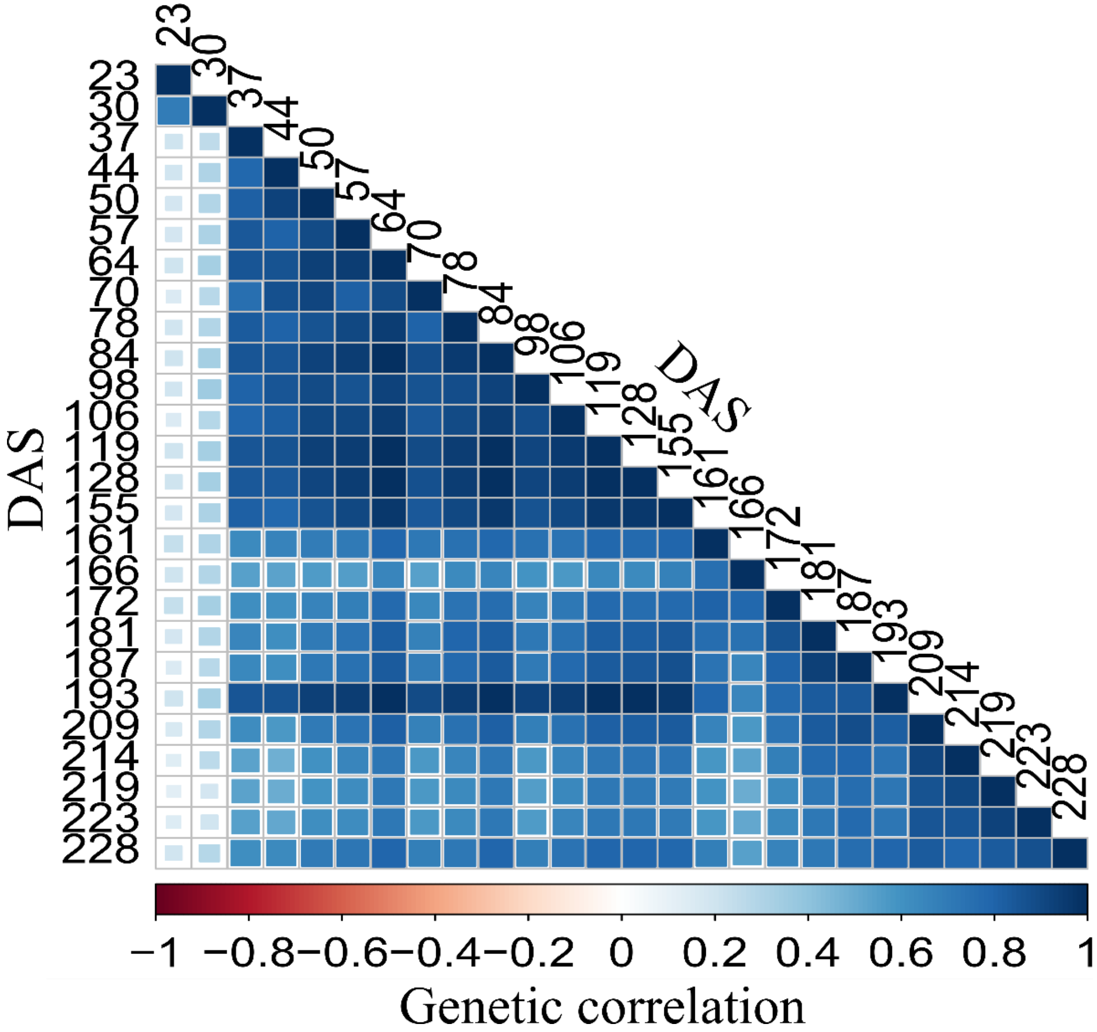

Supplementary Fig. S3. Heatmap of genetic correlations of height between 26 timepoints in 2017 data. DAS: days after sowing.

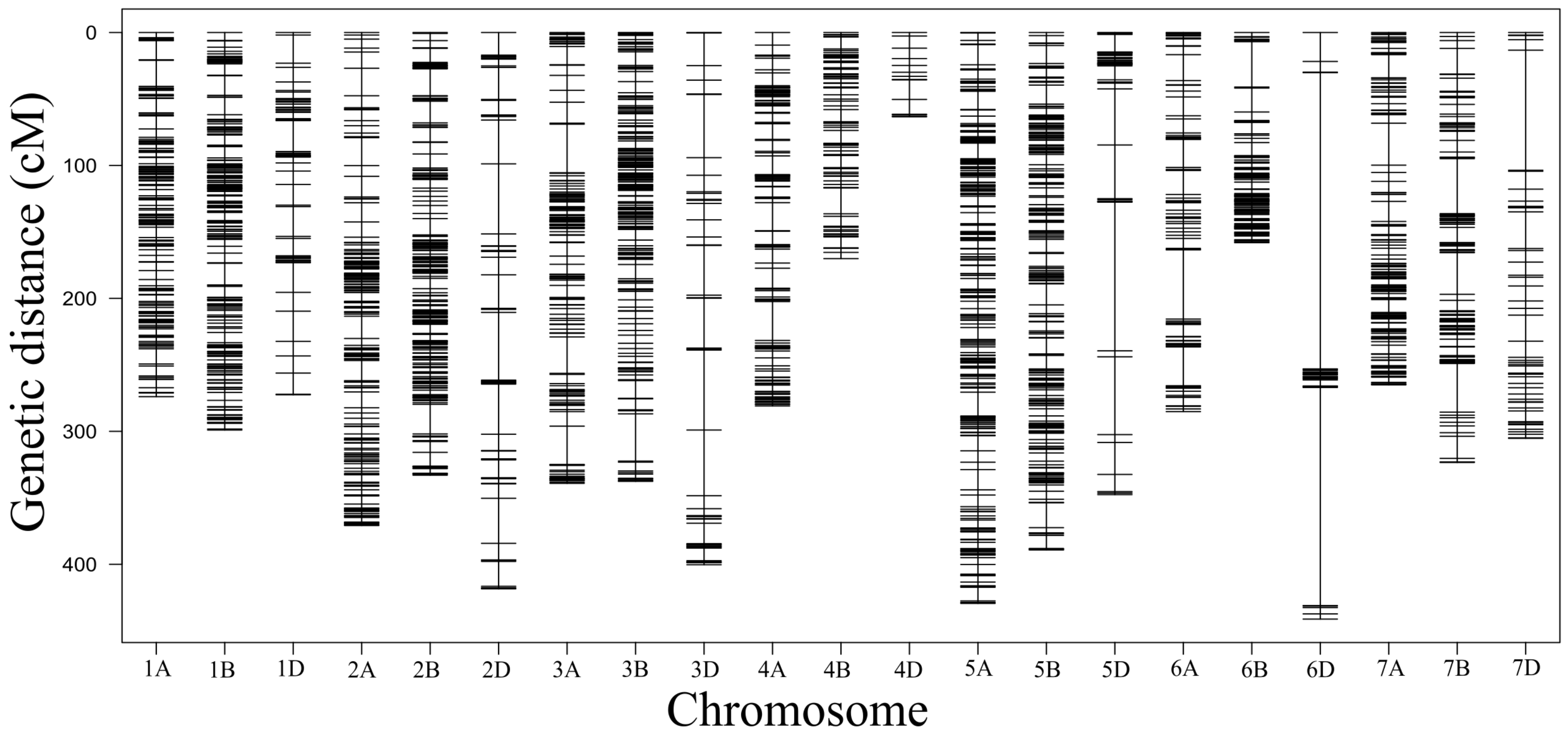

Supplementary Fig. S4. Genetic map consisting of 2330 SNPs. Map distances are shown in centimorgans (cM).

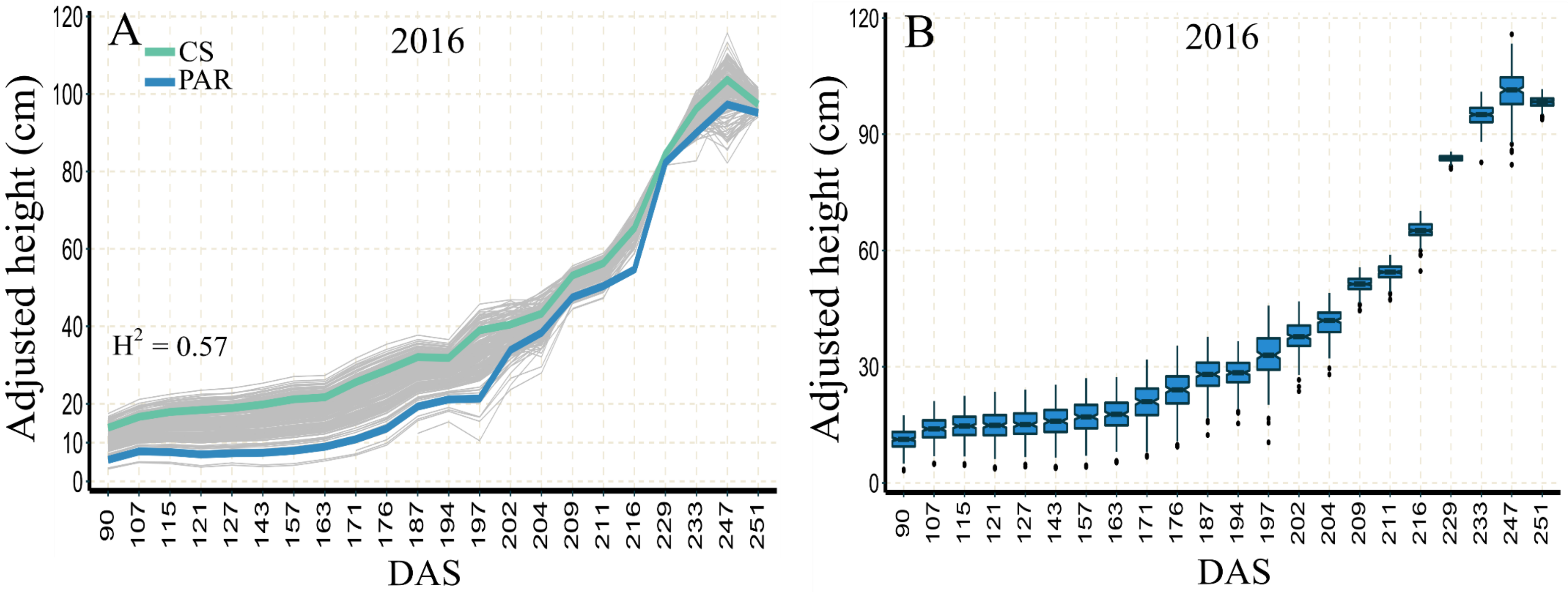

Supplementary Fig. S5. Temporal pattern of canopy height growth in a wheat recombinant mapping population measured from a robotic Field Scanalyzer in 2016. (A, B) Adjusted phenotypic values from 197 recombinant inbred line genotypes (grey lines), Chinese Spring (CS) and Paragon (PAR) parents (light green and blue, respectively) from 22 timepoints (TPs). The mean broad-sense (H^2) heritability across all TPs is shown inside the plot. DAS: days after sowing.

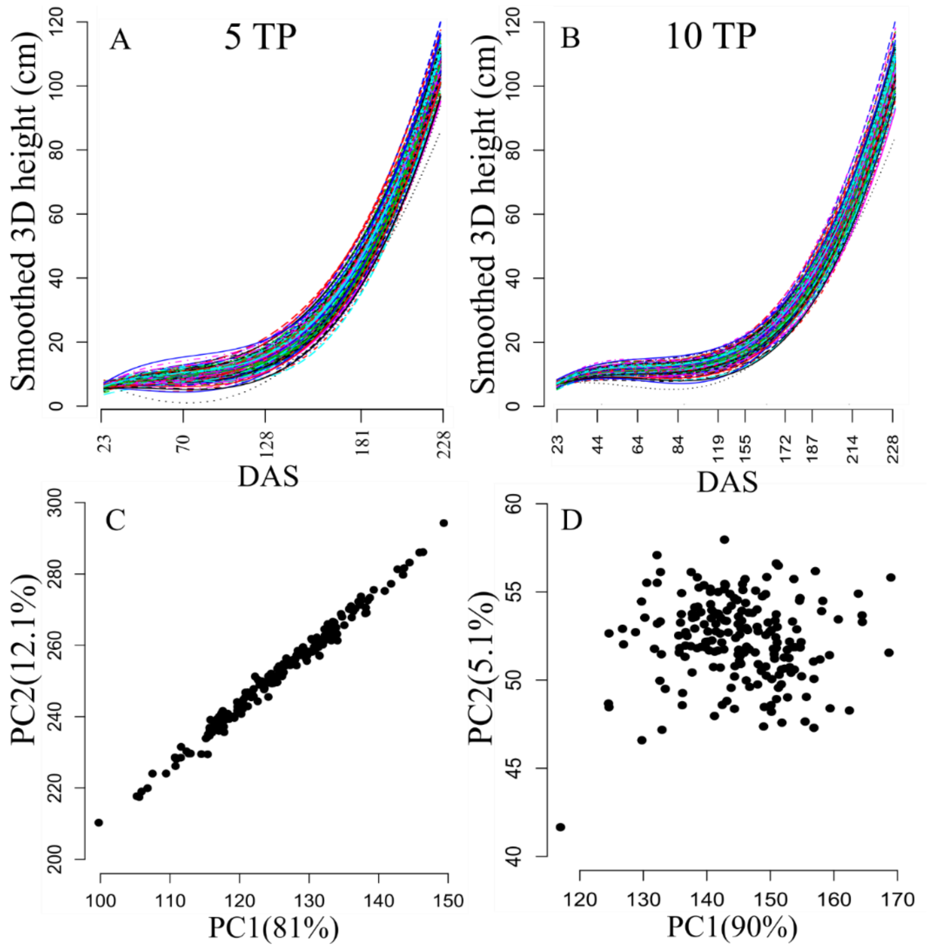

Supplementary Fig. S6. Smoothed and dimension-reduced height phenotypes in a recombinant mapping population of wheat in 2017. B-spline smoothed (A-B) and dimension-reduced [first (PC1) and second (PC2) functional principal components (C-D)] height phenotypes at five and 10 timepoints (TPs), respectively. Percentages in parentheses represent the variance explained by each of the two PCs. Each colored line or black dot represents the smoothed phenotype or PC1/PC2 of a recombinant inbred line genotype. The five- and 10-TP data correspond to the systematic R_1 and R_5 scenarios (see Table 1). DAS: days after sowing.

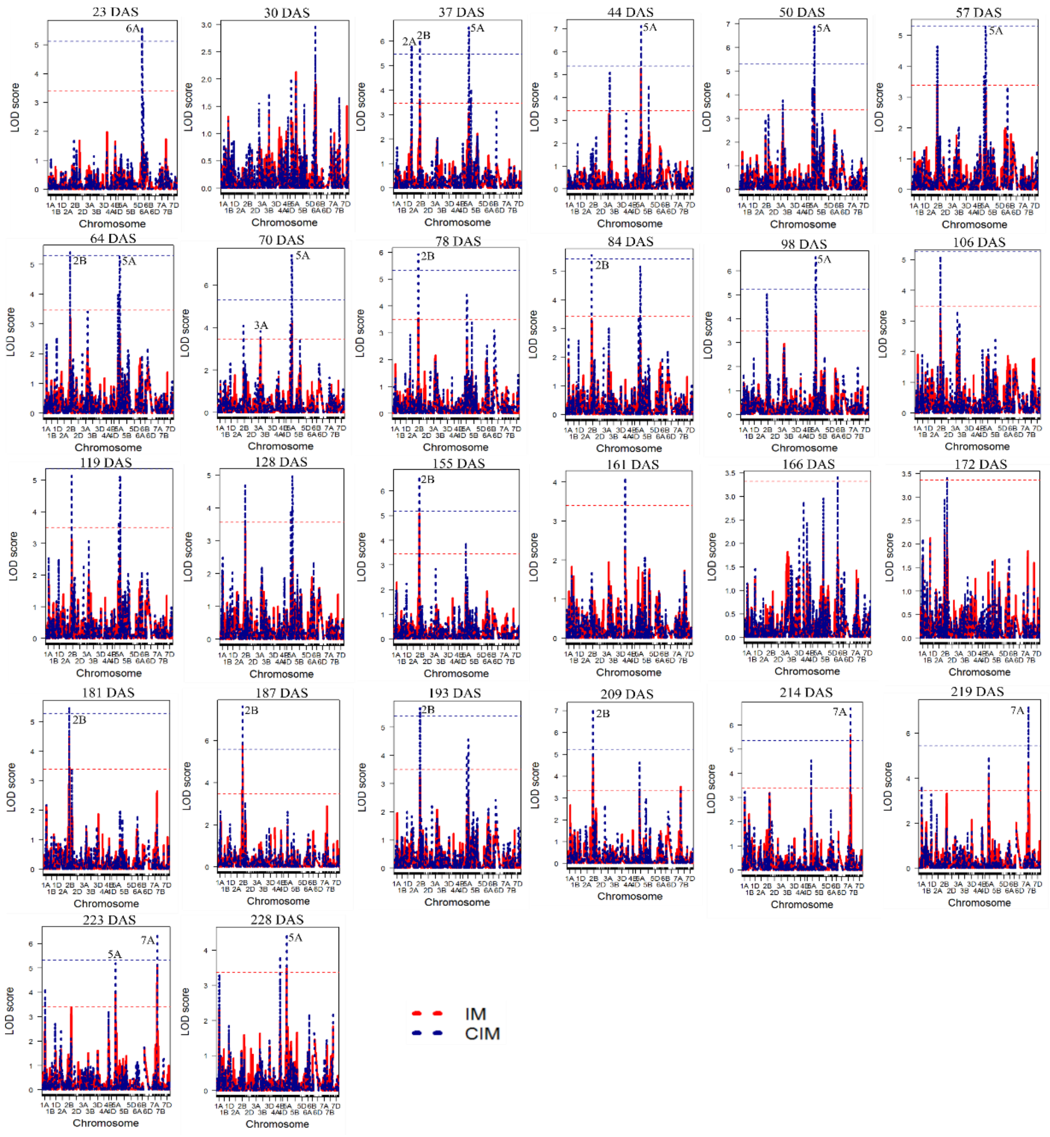

Supplementary Fig. S7. Interval (IM) and composite (CIM) QTL mapping for height at 26 timepoints in 2017. Dashed horizontal lines indicate the α = 0.05 permutation-based thresholds. DAS: days after sowing.

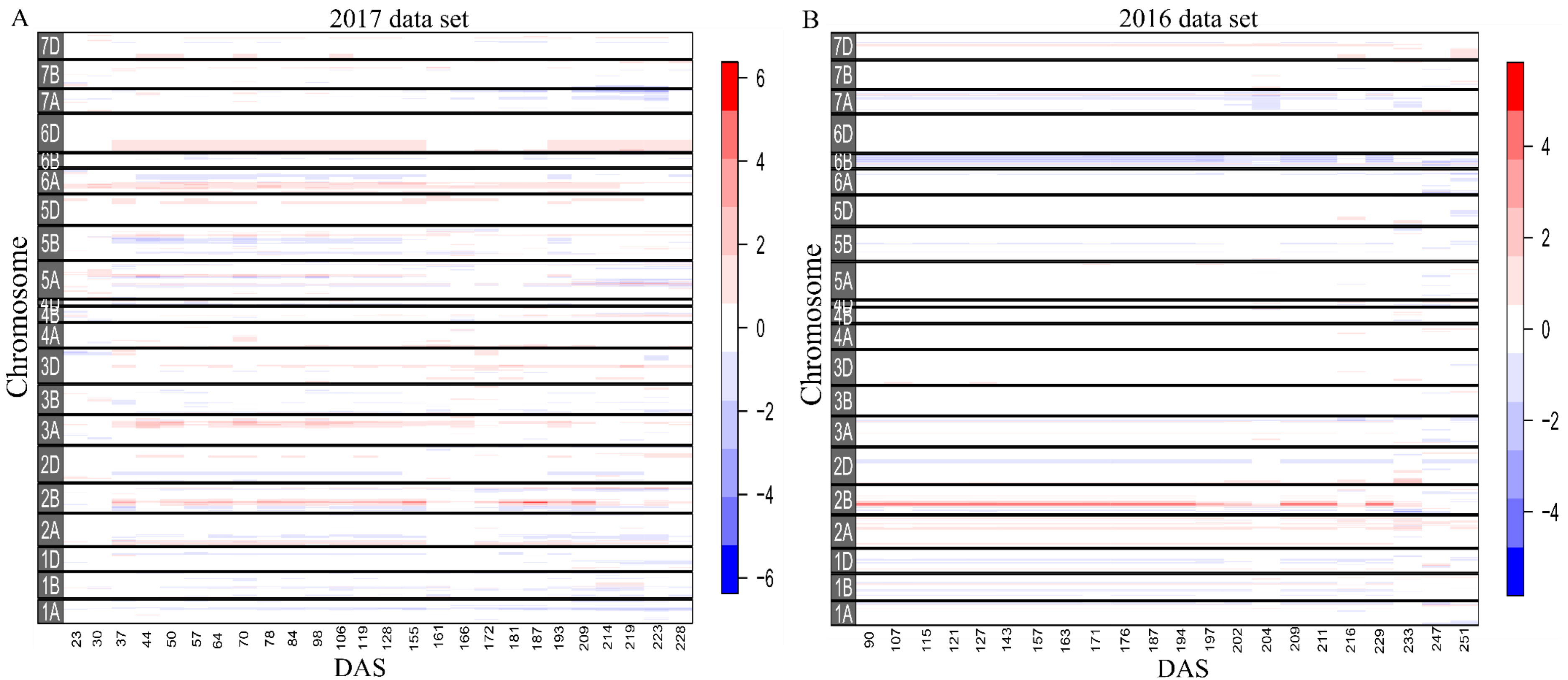

Supplementary Fig. S8. Signed *LOD* scores using the interval mapping (IM) with each timepoint (TP) considered individually for 2017 and 2016 data sets. Red: positive allele effect; Blue: negative allele effect. DAS: days after sowing.

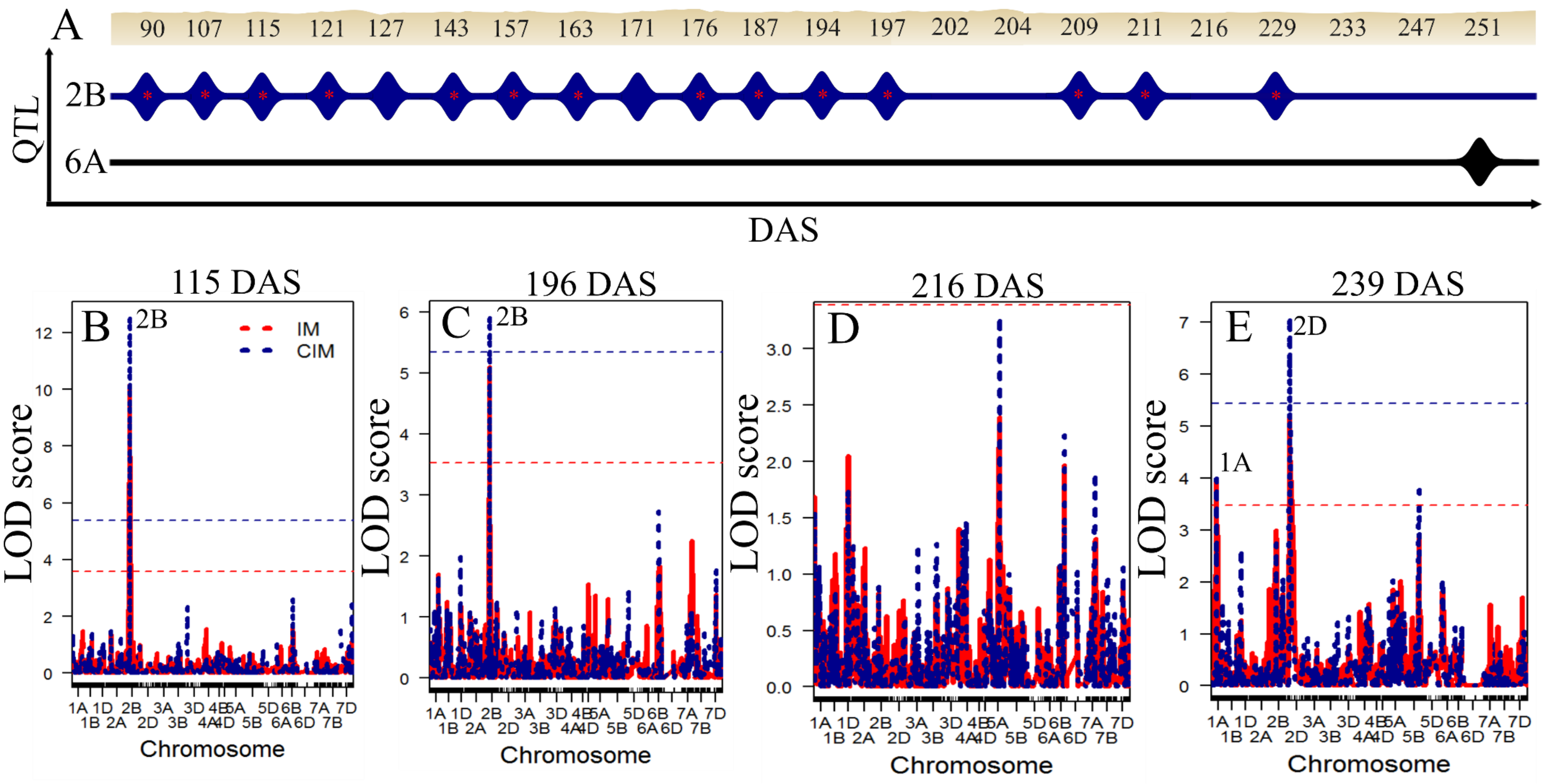

Supplementary Fig. S9. Summary of individual-timepoint (TP) mapping results in 2016. (A) Schematic representation showing the respective QTL detected at specific chromosomes in the individual mapping at each TP using interval and composite interval mapping for height from 22 TPs. Asterisks (*) indicate QTL identified only through interval mapping. (B-E) Interval (IM, dotted red line) and composite intevral (CIM, dotted blue line) QTL mapping for the manual plant height data at DAS 115, 196, 216, and 239. Dashed horizontal lines indicate α = 0.05 permutation-based thresholds. DAS: days after sowing.

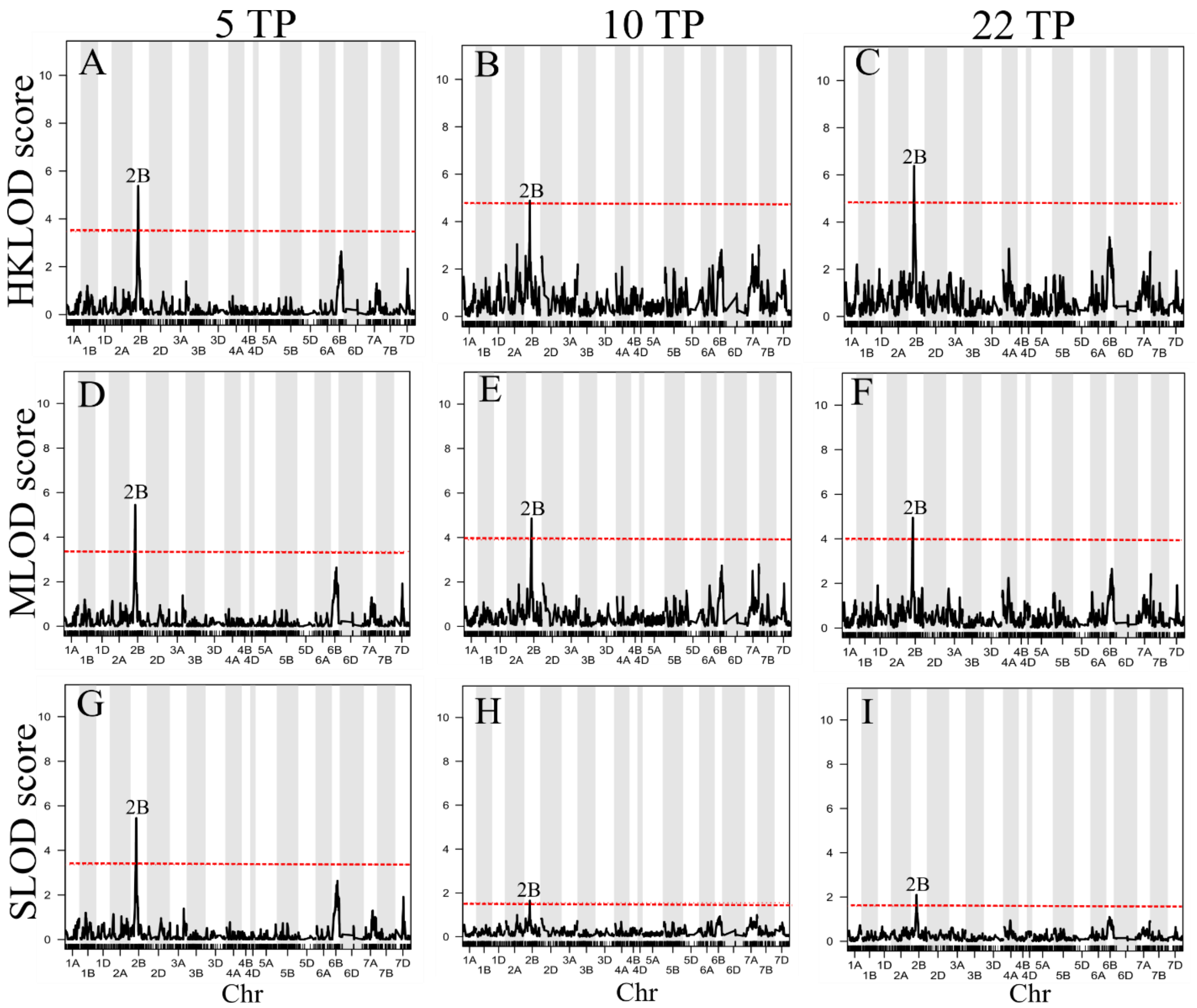

Supplementary Fig. S10. Functional QTL mapping analysis for five, 10, and 22 timepoints (TPs) in 2016. (A-I) Single functional QTL mapping model based on multi-trait (HKLOD), average (SLOD) and maximum (MLOD) profiles. Red horizontal lines indicate the α = 0.05 permutation-based thresholds.

Supplementary Table S1. Likelihood ratio test (LRT) for random effects and Wald test (WT) for fixed effects in 26 timepoints (TPs).

TPs	Variation source	
	Genotype	Check
23	50.28**	3.30 NS
30	35.88**	5.5 NS
37	104.46**	10.3 NS
44	42.04**	11.3 NS
50	52.21**	11.7 NS
57	40.24**	15.9 NS
64	29.57**	14.7 NS
70	86.51**	18.4*
78	48.97**	17.4*
84	30.32**	21.5*
98	42.12**	12.9 NS
106	22.35**	12.9 NS
119	66.61**	15.0 NS
128	49.25**	15.4 NS
155	56.81**	14.9 NS
161	76.31**	14.66 NS
166	114.90**	5.42 NS
172	98.79**	4.13 NS
181	78.44**	7.3 NS
187	34.04**	9.3 NS
193	11.55**	5.9 NS
209	34.53**	16.1*
214	61.82**	19.2*
219	44.39**	23.0**
223	40.72**	25.7**
228	10.89**	48.9**

Significant at * $\alpha = 0.05$; ** $\alpha = 0.01$ by LRT and WT; NS non-significant
